# Association of US Households’ Disaster Preparedness With Socioeconomic Characteristics, Composition, and Region

**DOI:** 10.1001/jamanetworkopen.2020.6881

**Published:** 2020-04-27

**Authors:** Lucila M. Zamboni, Erika G. Martin

**Affiliations:** 1 Center for Policy Research, Rockefeller College of Public Affairs and Policy, University at Albany-State University of New York, Albany; 2 Department of Public Administration and Policy, Rockefeller College of Public Affairs and Policy, University at Albany-State University of New York, Albany

## Abstract

**Importance:**

Since the terrorist attacks on September 11, 2001, the US government has promoted household disaster preparedness, but preparedness remains low.

**Objective:**

To identify disparities in disaster preparedness among US households.

**Design, Setting, and Participants:**

This cross-sectional study used data from a nationally representative sample of US households from the 2017 American Housing Survey’s topical section on preparedness to assess associations of disaster preparedness with households’ socioeconomic characteristics, composition, and region. Logistic regressions were used to assess associations of household characteristics with overall preparedness, resource- and action-based preparedness, and specific preparedness items. Data analyses were completed on March 27, 2020.

**Exposures:**

Combined household income, head of household’s education level, race/ethnicity, marital status of head of household, head of household aged 65 years or older, presence of children or a household member with a disability, and region.

**Main Outcomes and Measures:**

Nine actionable preparedness items, such as having an emergency carry-on kit (resource), food and water stockpiles (resource), and alternative communication plans and meeting locations (action). Items were summed for the measures of overall, resource-based, and action-based preparedness, with preparedness defined as meeting at least half of the criteria.

**Results:**

Among 16 725 included households, 9103 household heads were men (54.4%), 11 687 were married (69.9%), and 10 749 (66.1%) had some college education or higher. In all, 1969 household heads (11.8%) were black, while 2696 were Hispanic/Latino (16.1%); 3579 household heads (21.4%) were 65 years or older. A total of 7163 households (42.8%) included children, and 3533 households (21.2%) included a person with a disability. Households were more likely to fulfill at least half of the criteria for resource-based preparedness (10 950 households [65.5%]) than for action-based preparedness (6876 households [41.1%]). Wealthy households and those with household heads aged 65 years or older were more likely to fulfill at least half of resource-based items (wealthy households: adjusted odds ratio [aOR] by logged income, 1.18 [95% CI, 1.13-1.22]; household heads age ≥65 years: aOR, 1.42 [95% CI, 1.29-1.55) but less likely to fulfill action-based items (wealthy households: aOR: 0.96 [95% CI, 0.93-0.99]; household heads age ≥65 years: aOR, 0.92 [95% CI, 0.84-0.99]). Households with black household heads were more likely to fulfill items directly related to emergencies (carry-on emergency kit: aOR, 1.26 [95% CI, 1.14-1.39]; alternative communication plan: aOR, 1.55 [95% CI, 1.39-1.72]; alternative meeting location: aOR, 1.18 [95% CI, 1.07-1.31]) but less likely to fulfill resource-based items (at least half of resource items: aOR, 0.89 [95% CI, 0.80-0.99]).

**Conclusions and Relevance:**

These findings suggest that types of preparedness vary by household characteristics. Targeted strategies are needed to promote preparedness across communities.

## Key Points

**Question:**

What characteristics are associated with resource- and action-based disaster preparedness among US households?

**Findings:**

In this cross-sectional study using nationally representative survey data from 16 725 US households, 68.9% of households fulfilled at least half of recommended preparedness items, but households were more likely to fulfill resource- than action-based items. Fulfillment of specific resource- or action-based items differed substantially by household characteristics.

**Meaning:**

These findings suggest that because households may treat preparedness items differently, targeted strategies are needed to promote preparedness across communities, and risk communication should emphasize the importance of both resource- and action-based preparedness.

## Teaser

This cross-sectional study assesses the associations of household demographic, socioeconomic, and composition characteristics with the likelihood of meeting disaster preparedness criteria.

## Introduction

Since the terrorist attacks of September 11, 2001, the Federal Emergency Management Agency, the Centers for Disease Control and Prevention, and state and local governments have promoted household disaster preparedness, with a message of being informed, making plans, and having emergency kits.^1,2,3^ Climate change and extreme weather events have numerous effects on population health,^4^ making household preparedness important beyond terrorist attacks. Despite these efforts, studies have consistently found that US households are not ready for emergencies, there have been no significant improvements in preparedness, and the proportion of people fulfilling some actions, such as having a communication plan, has decreased.^5,6^ Even when individuals claim to be prepared, often they are not. The Centers for Disease Control and Prevention estimates that nearly 50% of US households do not have resources or plans in the event of emergency.^7^ In a separate study, the Federal Emergency Management Agency found that 70% of survey respondents completed at least 1 mitigation action but only 25% of respondents completed 2 mitigation actions. Although individuals may consider preparedness actions, their actions may be insufficient.^6^

High socioeconomic status has been consistently found to be associated with preparedness, but there are conflicting findings regarding other demographic characteristics. Higher socioeconomic status increases households’ odds of being prepared,^5,8,9,10,11^ and the likelihood of evacuating when required.^12,13^ However, results for racial and ethnic minority households are contradictory: some studies have found that these groups encounter more challenges to being prepared,^14,15^ while others have found that they are more likely to have emergency plans and supplies.^16^ Results also differ among households with individuals with disabilities, with some studies reporting that these households are less likely to be prepared^17,18^ and others reporting that they have higher odds of having an alternative meeting location.^16,19^ A limitation of these studies is that they only analyzed a few specific preparedness items, such as complying with mandatory evacuations,^12^ or examined emergency supplies and emergency plans in general.^6,8,11,16^

In this study, we used a nationally representative sample from the 2017 American Housing Survey to identify factors associated with household emergency preparedness and whether they differ by type of preparedness. We differentiated resource- vs action-based preparedness. Resource-based items are material or financial resources, such as food and water stockpiles, an electric generator, or having financial resources for evacuation. Action-based preparedness items are activities that enhance households’ preparedness through coordination actions, such as having an alternative communication plan or meeting location. These items do not rely on material resources and thus are less likely to have economic limitations for fulfillment. Previous studies have identified factors associated with some preparedness actions; however, to our knowledge, no study has analyzed extensively their associations with different levels and types of disaster preparedness.^5,9,10,11,16,18,20,21,22,23^ Taking this granular approach to examining preparedness is important because the relative importance of items may vary across disasters. For example, having emergency preparedness kits, financial resources, food stockpiles, and communication plans are important for responding to the novel coronavirus pandemic owing to temporary shelter in place restrictions and associated economic loss, whereas water stockpiles, electronic generators, an alternative meeting point, and a vehicle available for evacuation are relevant to responding to a natural disaster.

## Methods

### Study Overview and Data Sources

Our cross-sectional study used household-level data from the nationally representative 2017 American Housing Survey (AHS), the most recent wave. The public use file microdata are available for direct public download on the US Census Bureau website. The AHS website includes the complete codebook and the interview script with details on participant consent, including the survey purpose and confidentiality assurances.^24^ Following the American Association for Public Opinion Research (AAPOR) reporting guideline,^25^ the survey website also contains details on accuracy, recruitment, and historical changes to questionnaires.^26^ We received an exemption from institutional review board review and informed consent from the University at Albany because this is a secondary data analysis. We tested the associations of overall disaster preparedness and different types of preparedness with households’ socioeconomic characteristics, composition, and region.

Although 66 752 housing units were included in the 2017 AHS, our analysis includes 33 474 occupied housing units included in the survey’s topical section on emergency and disaster preparedness. Households included in our sample responded to all 9 questions on emergency and disaster preparedness used as our outcome measures. eTable 1 in the Supplement compares demographic characteristics of survey participants included and excluded from our study.

### Variables

The survey contains 12 preparedness items; following past studies, we analyze the 9 items that are actionable by household members.^18^ eTable 2 in the Supplement lists all survey questions, defining each included resource-based item (ie, vehicle available for evacuation, food stockpiles, financial resource for evacuation, water stockpiles, carry-on emergency preparedness kit, and electric generator) and action-based item (ie, plan with financial information, separate evacuation meeting point, and alternative communication plan). The 3 excluded items are not readily actionable: assistance needed to evacuate or shelter pets, home has a tornado safe room or shelter, and whether a disaster in the past 2 years required the household or landlord to make extensive home repairs.

Consistent with previous studies, the values for all 9 items were summed for our measure of overall preparedness, coded as a binomial indicator for which preparedness was defined as meeting more than half of the criteria.^17,18,20^ For the resource- and action-based preparedness measures, the 9 items were first classified by type of preparedness and then summed within each group. Households were classified as prepared if they fulfilled at least half of the items. We selected that cutoff because it was a midpoint to distinguish levels of preparedness, having all regressions in the same logistic format allows for an easier interpretation, and sensitivity analyses using ordinary least squares on a continuous 0 to 9 outcome and a multinomial logit for low, medium, and high levels of preparedness yielded similar findings (eTable 3 in the Supplement). We did not weight preparedness items differently in our indices because their comparative importance and effectiveness has not been demonstrated.^20,27^

The independent variables were socioeconomic factors previously identified as influencing household or individual disaster preparedness and region. Included socioeconomic measures were combined household income (log-transformed) and the head of household’s education level.^6,9,11,16,18,21,22,28^ To assess whether the income relationship was curvilinear, we also examined income as quadratic and as quintiles, and results were similar (eTable 4 in the Supplement). Included household composition measures were race/ethnicity (black and Hispanic/Latino), woman head of household, whether the head of the household was married, whether the household included an individual with a disability, whether the head of the household was 65 years or older, and the presence of children younger than 18 years.^14,15,16,17,18,19,22,23,29,30^ Race and ethnicity were self-reported by respondents using the standard Census definitions, and for black race we included those self-disclosing as their primary identity. We focus on heads of households 65 years or older because older populations are commonly identified as being at higher risk, and this cutoff is consistent with past studies.^17,20,31^ The household’s geographical region was operationalized as 3 dummy variables for Northeast, Midwest, and West regions, with the South region as the reference.^5,6,22^ Urbanicity was not included owing to its unavailability in the public use file. There was low correlation among all independent variables, reducing multicollinearity concerns with the adjusted models.

### Statistical Analysis

Three logistic regression models tested the associations of household characteristics with fulfilling at least half of the items for overall preparedness, resource-based preparedness, and action-based preparedness. Additionally, 9 logistic regression models examined the associations of household-level characteristics with each preparedness item as dependent variables to assess consistency in the statistical significance and direction of the household characteristics across preparedness items. We report 95% CIs rather than *P* values owing to the large number of associations considered. All statistical analyses were conducted using SAS statistical software version 9.4 (SAS Institute).

We did not use the survey weights because our study sample was restricted to individuals who answered all 9 preparedness questions. Qualitative conclusions from frequencies and regressions using a logistic regression weighting to adjust for nonresponse were similar (eTables 5, 6, 7, and 8 in the Supplement). Data analyses were completed on March 27, 2020.

## Results

Of 33 474 households that received the 2017 AHS disaster preparedness module, 16 725 households responded to all preparedness questions and were included in our sample (50.0% participation rate). Self-reported characteristics of household heads included 9103 (54.4%) men, 11 687 household heads (69.9%) were married, 1969 were black (11.8%), 2696 (16.1%) were Hispanic/Latino, 3579 (21.4%) were aged 65 years or older, and 11 049 (66.1%) had some college or higher education (Table 1). Household characteristics included 7163 households (42.8%) with children and 3533 households (21.2%) with a person with a disability. Median (interquartile range) annual household income was $76 400 ($40 700-$128 100).

**Table 1.** Household Characteristics and Fulfillment of Preparedness Items

Characteristic	No. (%)
**Head of household**	
Men	9103 (54.4)
Married	11 687 (69.9)
Race/ethnicity	
Black	1969 (11.8)
Hispanic/Latino	2696 (16.1)
Age ≥65 y	3579 (21.4)
Education level	
No high school degree	1772 (10.6)
High school degree	3904 (23.3)
Some college	8497 (50.8)
Some graduate education	2552 (15.3)
**Household**	
Combined income, median (IQR), $	76 400 (40 700-128 100)
Presence of children	7163 (42.8)
Presence of a person with a disability	3533 (21.2)
Census region	
South	6469 (38.7)
Northeast	2445 (14.6)
West	4568 (27.3)
Midwest	3243 (19.4)
**Preparedness**	
Resource-based items	
Vehicle available for evacuation	16 087 (96.2)
Food stockpile	14 018 (83.8)
Financial resources for evacuation	13 505 (80.8)
Water stockpile	10 000 (59.8)
Carry-on emergency preparedness kit	9166 (54.8)
Electric generator	3167 (18.9)
Action-based items	
Plan with financial information	13 800 (82.5)
Separate evacuation meeting point	6381 (38.2)
Alternative communication plan	4670 (27.9)
Fulfillment of preparedness criteria^a^	
Overall	11 522 (68.9)
Resource-based	10 950 (65.5)
Action-based	6876 (41.1)

Abbreviation: IQR, interquartile range.

^a^ Preparedness is defined as meeting at least half of the items in that category.

Households fulfilled a variable number of preparedness items (Figure). More than two-thirds of households (11 522 households [68.9%]) met the criteria for overall preparedness, defined as meeting at least half of the criteria, with approximately half of households (9009 households [53.9%]) meeting 4, 5, or 6 items. Only 579 households (3.5%) met all 9 items. However, preparedness differed by type of action. A total of 10 950 households (65.5%) fulfilled at least half of resource-based preparedness items, while 6876 households (41.1%) fulfilled at least half of action-based preparedness items. Most households had food stockpiles (14 018 households [83.8%]), vehicles available for evacuation (16 087 households [96.2%]), and financial resources for evacuation (13 505 households [80.8%]). More than half of the households reported having water stockpiles (10 000 households [59.8%]) and carry-on emergency preparedness kits (9166 households [54.8%]), but a minority of households reported having electric generators (3167 households [18.9%]). Fulfilling action-based preparedness items was less common. Although 13 800 households (82.5%) had a plan with their financial information, only 6381 households (27.9%) had alternative communication plans and 6381 households (38.2%) had separate evacuation meeting points. Correlations for fulfillment of items is presented in eTable 9 in the Supplement.

**Figure.**
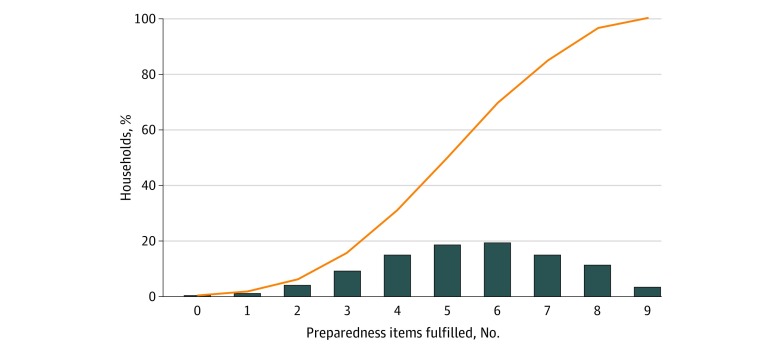
Distribution of US households' Fulfillment of Preparedness Items Bars indicate percentage of households fulfilling that number of items and the orange line, cumulative percentage.

In the adjusted models, household member characteristics and region were significantly associated with preparedness (Table 2). There was higher overall preparedness among households whose heads were married (adjusted odds ratio [aOR], 1.47 [95% CI, 1.36-1.60]), 65 years or older (aOR, 1.26 [95% CI, 1.15-1.39]), and with higher levels of education (compared with less than high school degree, high school degree: aOR, 1.35 [95% CI, 1.19-1.52]; some college: aOR, 1.39 [95% CI, 1.23-1.56]; some graduate education: aOR, 1.11 [95% CI, 0.96-1.28]) and among households with higher logged income (aOR, 1.13 [95% CI, 1.09-1.18]). Compared with households in the South, households in all other regions were less likely to meet criteria for overall preparedness (Northeast: aOR, 0.77 [95% CI, 0.70-0.85]; West: aOR, 0.87 [95% CI, 0.80-0.95]; Midwest: aOR, 0.83 [95% CI, 0.76-0.91]). There was lower overall preparedness among woman-headed households (aOR, 0.89 [95% CI, 0.83-0.96]), households with children (aOR, 0.87 [95% CI, 0.81-0.94]), and households with a person with a disability (aOR, 0.86 [95% CI, 0.79-0.94]).

**Table 2.** Logistic Regression of Adjusted Odds of Preparedness Among US Households Stratified by Type of Preparedness

Characteristic	Preparedness, aOR (95% CI)^a^		
	Overall	Resource-based	Action-based
**Head of household**			
Woman	0.89 (0.83-0.96)	0.85 (0.80-0.91)	0.96 (0.90-1.03)
Married	1.47 (1.36-1.59)	1.54 (1.43-1.66)	1.22 (1.14-1.32)
Race/ethnicity			
Black	0.99 (0.89-1.10)	0.89 (0.80-0.99)	1.25 (1.13-1.38)
Hispanic/Latino	0.88 (0.80-0.97)	0.92 (0.84-1.01)	0.95 (0.87-1.04)
Age ≥65 y	1.26 (1.15-1.39)	1.42 (1.29-1.55)	0.92 (0.84-0.99)
Education level			
No diploma	1 [Reference]	1 [Reference]	1 [Reference]
High school diploma	1.35 (1.19-1.52)	1.26 (1.12-1.42)	1.18 (1.04-1.33)
Some college	1.39 (1.23-1.56)	1.28 (1.14-1.43)	1.18 (1.06-1.33)
Some graduate education	1.11 (0.96-1.28)	1.09 (0.87-1.16)	1.07 (0.93-1.23)
**Household**			
Combined income, logged	1.13 (1.09-1.18)	1.18 (1.13-1.22)	0.96 (0.93-0.99)
Presence of children	0.87 (0.81-0.94)	0.75 (0.70-0.80)	1.24 (1.16-1.33)
Presence of a person with a disability	0.86 (0.79-0.94)	0.83 (0.76-0.90)	1.04 (0.96-1.23)
Location			
South	1 [Reference]	1 [Reference]	1 [Reference]
Northeast	0.77 (0.70-0.85)	0.84 (0.76-0.93)	0.76 (0.69-0.84)
West	0.87 (0.80-0.95)	0.95 (0.88-1.04)	0.92 (0.85-0.99)
Midwest	0.83 (0.76-0.91)	0.81 (0.74-0.90)	0.81 (0.74-0.89)

Abbreviation: aOR, adjusted odds ratio.

^a^ Preparedness is defined by fulfilling at least half of items.

There were differences in preparedness for resource- vs action-based items (Table 2). Households with higher logged incomes were more likely to fulfill at least half of resource-based items (aOR, 1.18 [95% CI, 1.13-1.22]) but less likely to fulfill at least half of action-based items (aOR, 0.96 [95% CI, 0.93-0.99]). Households whose head was black (aOR, 0.89 [95% CI, 0.80-0.99]) and households with children (aOR, 0.75 [95% CI, 0.70-0.80]) were less likely to fulfill the resource-based preparedness criterion but more likely to fulfill the action-based preparedness criterion (black household head: aOR, 1.25 [95% CI, 1.13-1.38]; households with children: aOR, 1.24 [95% CI, 1.16-1.33]). Woman-headed households (aOR, 0.85 [95% CI, 0.80-0.91]) and households with a person with a disability (aOR, 0.83 [95% CI, 0.76-0.90]) were less likely to fulfill at least half of resource-based items, but these characteristics were not associated with fulfilling at least half of action-based preparedness items.

Table 3 and Table 4 present the adjusted models for the adjusted odds of fulfilling specific resource- and action-based preparedness items. Many findings are consistent with the results from the aggregate preparedness measures in Table 2. For example, woman-headed households had lower preparedness on half of individual resource-based items, including having an electric generator (aOR, 0.89 [95% CI, 0.82-0.97]), a carry-on emergency kit (aOR, 0.88 [95% CI, 0.82-0.94]), and financial resources for evacuation (aOR, 0.73 [95% CI, 0.66-0.80]). Married household heads were more likely to fulfill each resource-based item (food stockpile: aOR, 1.20 [95% CI, 1.09-1.31]; water stockpile: aOR, 1.27 [95% CI, 1.18-1.37]; electric generator: aOR, 1.88 [95% CI, 1.69-2.08]; carry-on emergency kit: aOR, 1.20 [95% CI, 1.12-1.29]; financial resources for evacuation: aOR, 1.71 [95% CI, 1.55-1.88]; evacuation vehicle: aOR, 2.04 [95% CI, 1.70-2.46]) and action-based item (communication plan: aOR, 1.13 [95% CI, 1.04-1.23]; meeting location: aOR, 1.20 [95% CI, 1.11-1.30; plan with financial information: aOR, 1.26 [95% CI, 1.15-1.38]). However, there were differences in the associations between household demographic characteristics and the likelihood of fulfilling items reflecting financial security more generally (eg, financial resources for an evacuation) or was exclusive to emergencies. Households with heads who were black were more likely to fulfill specific resource- and action-based items that were related to emergencies but did not require substantial resources (water stockpile: aOR, 1.18 [95% CI, 1.07-1.31]; carry-on emergency kit: aOR, 1.26 [95% CI, 1.14-1.39]; alternative communication plan: aOR, 1.55 [95% CI, 1.39-1.72]; alternative meeting location: aOR, 1.18 [95% CI, 1.07-1.31]) but less likely to fulfill items requiring large investments or financial stability (food stockpile: aOR, 0.76 [95% CI, 0.66-0.86]; electric generator: aOR, 0.57 [95% CI, 0.49-0.67]; financial resources for evacuation: aOR, 0.54 [95% CI, 0.48-0.61]; evacuation vehicle: aOR, 0.41 [95% CI, 0.33-0.50]). Households whose heads were Hispanic/Latino had a similar pattern of lower preparedness on specific items requiring substantial resources (food stockpile: aOR, 0.68 [95% CI, 0.61-0.76]; electric generator: aOR, 0.64 [95% CI, 0.56-0.74]; financial resources: aOR, 0.66 [95% CI, 0.59-0.74]; evacuation vehicle: aOR, 0.57 [95% CI, 0.46-0.70]), but they were more likely to have a water stockpile (aOR, 1.45 [95% CI, 1.31-1.59]) and alternative communication plan (aOR, 1.12 [95% CI, 1.02-1.24]). Households with higher incomes were more likely to fulfill preparedness items requiring resources and reflecting financial stability (food stockpile: aOR, 1.09 [95% CI, 1.04-1.14]; electric generator: aOR, 1.19 [95% CI, 1.13-1.25]; financial resources for evacuation: aOR, 2.02 [95% CI, 1.92-2.13]; evacuation vehicle: aOR, 1.44 [95% CI, 1.34-1.55]; plan with financial information: aOR, 1.21 [95% CI, 1.16-1.27]), but they were less likely to have an alternative communication plan (aOR, 0.89 ([95% CI, 0.86-0.93]) or alternative meeting location (aOR, 0.96 [95% CI, 0.93-0.99]). Households whose heads had higher levels of education had increased odds of having financial resources for evacuation (high school diploma: aOR, 1.64 [95% CI, 1.43-1.88]; some college: aOR, 2.56 [95% CI, 2.24-2.93); some graduate education: aOR, 7.04 [95% CI, 5.51-8.99]), an evacuation vehicle (high school diploma: aOR, 1.66 [95% CI, 1.31-2.11]; some college: aOR, 2.03 [95% CI, 1.61-2.56]; some graduate education: aOR, 2.34 [95% CI, 1.61-3.40]), and a financial plan (high school diploma: aOR, 1.50 [95% CI, 1.31-1.72]; some college: aOR, 1.81 [95% CI, 1.59-2.06]; some graduate education: aOR, 1.79 [95% CI, 1.50-2.12]), but increasing levels of education were associated with decreasing odds of having a water stockpile (high school diploma: aOR, 0.88 [95% CI, 0.78-0.99]; some college: aOR, 0.80 [95% CI, 0.71-0.90]; some graduate education: aOR, 0.64 [95% CI, 0.55-0.74]).

**Table 3.** Logistic Regression of Adjusted Odds of Fulfillment of Resource-Based Preparedness Items Among US Households

Characteristic	aOR (95% CI)					
	Stockpile		Electric generator	Carry-on emergency kit	Financial resources for evacuation	Evacuation vehicle
	Food	Water				
**Head of household**						
Woman	0.99 (0.91-1.08)	0.95 (0.89-1.02)	0.89 (0.82-0.97)	0.88 (0.82-0.94)	0.73 (0.66-0.80)	0.95 (0.79-1.14)
Married	1.20 (1.09-1.31)	1.27 (1.18-1.37)	1.88 (1.69-2.08)	1.20 (1.12-1.29)	1.71 (1.55-1.88)	2.04 (1.70-2.46)
Race/ethnicity						
Black	0.76 (0.66-0.86)	1.18 (1.07-1.31)	0.57 (0.49-0.67)	1.26 (1.14-1.39)	0.54 (0.48-0.61)	0.41 (0.33-0.50)
Hispanic/Latino	0.68 (0.61-0.76)	1.45 (1.31-1.59)	0.64 (0.56-0.74)	0.97 (0.89-1.06)	0.66 (0.59-0.74)	0.57 (0.46-0.70)
Age ≥65 y	1.38 (1.22-1.56)	1.27 (1.17-1.39)	1.16 (1.05-1.29)	1.02 (0.94-1.11)	2.35 (2.05-2.69)	1.15 (0.92-1.44)
Education level						
No diploma	1 [Reference]	1 [Reference]	1 [Reference]	1 [Reference]	1 [Reference]	1 [Reference]
High school diploma	1.26 (1.08-1.46)	0.88 (0.78-0.99)	1.12 (0.96-1.31)	1.15 (1.02-1.29)	1.64 (1.43-1.88)	1.66 (1.31-2.11)
Some college	1.23 (1.06-1.41)	0.80 (0.71-0.90)	0.91 (0.78-1.07)	1.10 (0.98-1.23)	2.56 (2.24-2.93)	2.03 (1.61-2.56)
Some graduate education	1.13 (0.94-1.34)	0.64 (0.55-0.74)	0.59 (0.49-0.71)	0.96 (0.83-1.10)	7.04 (5.51-8.99)	2.34 (1.61-3.40)
**Household**						
Combined income, logged	1.09 (1.04-1.14)	0.97 (0.93-1.00)	1.19 (1.13-1.25)	1.01 (0.97-1.05)	2.02 (1.92-2.13)	1.44 (1.34-1.55)
Presence of children	0.92 (0.84-1.00)	0.72 (0.67-0.77)	0.74 (0.68-0.81)	0.92 (0.86-0.98)	0.68 (0.62-0.75)	1.06 (0.88-1.27)
Presence of a person with a disability	0.98 (0.88-1.10)	0.88 (0.81-0.95)	1.23 (1.11-1.36)	0.92 (0.85-1.00)	0.55 (0.49-0.61)	0.59 (0.49-0.71)
Location						
South	1 [Reference]	1 [Reference]	1 [Reference]	1 [Reference]	1 [Reference]	1 [Reference]
Northeast	0.99 (0.87-1.13)	0.92 (0.83-1.01)	1.04 (0.92-1.17)	0.68 (0.62-0.75)	1.09 (0.95-1.26)	0.42 (0.33-0.52)
West	0.82 (0.74-0.91)	1.16 (1.07-1.26)	0.65 (0.59-0.73)	0.96 (0.88-1.04)	1.07 (0.95-1.20)	0.89 (0.71-1.12)
Midwest	1.03 (0.92-1.17)	0.81 (0.74-0.89)	1.03 (0.93-1.15)	0.66 (0.60-0.72)	1.03 (0.91-1.16)	1.00 (0.77-1.29)

Abbreviation: aOR, adjusted odds ratio.

**Table 4.** Logistic Regression of Adjusted Odds of Fulfillment of Action-Based Preparedness Items Among US Households

Characteristic	aOR (95% CI)		
	Alternative communication plan	Alternative meeting location	Plan with financial information
**Head of household**			
Female	0.96 (0.89-1.03)	0.97 (0.91-1.03)	0.93 (0.85-1.01)
Married	1.13 (1.04-1.23)	1.20 (1.11-1.30)	1.26 (1.15-1.38)
Race/ethnicity			
Black	1.55 (1.39-1.72)	1.18 (1.07-1.31)	0.98 (0.86-1.12)
Hispanic/Latino	1.12 (1.02-1.24)	0.92 (0.84-1.01)	0.72 (0.65-0.81)
Age ≥65 y	0.91 (0.83-1.00)	0.85 (0.78-0.93)	1.10 (0.99-1.23)
Education level			
No diploma	1 [Reference]	1 [Reference]	1 [Reference]
High school diploma	1.12 (0.99-1.28)	1.16 (1.03-1.31)	1.50 (1.31-1.72)
Some college	1.01 (0.89-1.15)	1.18 (1.05-1.33)	1.81 (1.59-2.06)
Some graduate education	0.96 (0.83-1.12)	1.05 (0.91-1.20)	1.79 (1.50-2.12)
**Household**			
Combined income, logged	0.89 (0.86-0.93)	0.96 (0.93-0.99)	1.21 (1.16-1.27)
Presence of children	1.08 (1.00-1.16)	1.28 (1.19-1.37)	0.94 (0.86-1.03)
Presence of a person with a disability	1.01 (0.92-1.10)	1.04 (0.96-1.13)	0.82 (0.74-0.91)
Location			
South	1 [Reference]	1 [Reference]	1 [Reference]
Northeast	0.77 (0.69-0.85)	0.81 (0.73-0.89)	0.70 (0.62-0.79)
West	1.07 (0.98-1.16)	0.96 (0.88-1.04)	0.69 (0.62-0.77)
Midwest	0.76 (0.68-0.83)	0.93 (0.85-1.02)	0.76 (0.67-0.85)

Abbreviation: aOR, adjusted odds ratio.

## Discussion

This cross-sectional study used recent nationally representative survey data to test the associations of household characteristics (ie, socioeconomic characteristics, composition, and region) with disaster preparedness (ie, overall preparedness, resource- and action-based preparedness, and specific preparedness items). Separating these items allows for a more nuanced understanding of which households are the highest risk for different types of disasters. Most households in our sample had some level of preparedness, but they were more likely to meet at least half of resource-based items (65.5% of households) than at least half of action-based items (41.1% of households). Types of preparedness varied by household characteristics; for example, wealthier households and those with adults 65 years or older were more likely to meet resource-based items but less likely to meet action-based items, households with children were generally less prepared but more likely to have an alternative meeting location, and households with black household heads were more likely to meet items directly related to emergencies but less likely to meet resource-based items that require large financial investments or financial stability.

Our study adds to the findings of prior studies on the determinants of preparedness and resilience by categorizing preparedness items as resource- or action-based preparedness. For example, a key finding of our study is that households with higher combined income had higher odds of having resource-based preparedness that requires large investments or financial stability, but we found no evidence of among these households of differences in fulfilling resource-based items specific to emergencies and lower odds of having action-based preparedness. Households with children were less likely to fulfill resource-based preparedness items and more likely to fulfill action-based preparedness items. These households may have had less disposable income available for resource-based items, but having an alternative communication plan and an alternative meeting location may have been a spillover effect from managing households with children where these may be school requirements. Another striking change in the direction of the association by type of preparedness occurred among black households. These households had lower odds of fulfilling at least half of resource-based preparedness items but increased odds of fulfilling at least half of action-based preparedness items. The shifts in the direction of the association were more evident when considering each individual preparedness item, with differences in these households’ odds of fulfilling resource-based items requiring larger financial investments (eg, electric generators, financial resources evacuation, and evacuation vehicle) vs resource-based items that are specific to emergency preparedness (eg, water stockpiles and carry-on emergency kits).

Previous studies analyzing the associations of household socioeconomic characteristics with disaster preparedness are contradictory at times. Our approach to classify items into resource- vs action-based measures might explain how different studies could be reaching conflicting conclusions. Previous studies have consistently found that determinants of higher socioeconomic status, such as higher levels of income^6,9,10,11^ or education,^6,8,12^ are positively associated with disaster preparedness. Our findings are consistent with these results. However, our study found that more financially secure households had reduced odds of having action-based disaster preparedness items that require coordination among household members and had a similar likelihood of having emergency supplies, such as water stockpiles and carry-on emergency kits. This suggests that while households’ financial stability may reduce the effect of a disaster, these households may face bigger challenges if disasters hit while they are away from home.

As they may require assistance during emergencies, households with persons with disabilities may be at increased risk. We found that this population was overall less prepared, particularly in their limited fulfillment of resource-based items, such as water stockpiles, financial resources for evacuation, and access to evacuation vehicles. We found no evidence of a positive association among these households with having an alternative meeting location, as previous studies have shown.^16,19^ Nevertheless, identifying the challenges this specific population faces when it comes to evacuation capability is critical to plan emergency response actions.

There are a few possible explanations to support these findings. First, households may have different perceptions of which preparedness items are relevant or have varying capacities to be prepared in different areas. For example, lower-income households or those with children may have fewer liquid assets to purchase resource-based items, and higher-income households may underestimate their likelihood of being affected by a disaster and the importance of action-based items. Second, while it is promising that many households met several of the recommended preparedness actions, the items frequently met are not exclusive to disaster preparedness. Having stockpiles available, evacuation vehicles, or financial resources for evacuation may not be the result of explicit disaster preparedness actions but evidence of general consumption patterns. If households are only preparing for the critical first 72 hours, we would expect to find that food and water stockpiles had similar association with household-level factors, but this was not the case. For example, the associations of racial/ethnic minority populations with their likelihoods of fulfilling food and water stockpiles were significant but in opposite directions. Our finding that households with children had a positive association with action-based preparedness despite having lower resource-based preparedness may indicate a spillover effect from school-based requirements. While we cannot confirm this, it may be a starting point to explore the influence schools may have in promoting disaster and emergency preparedness.

There are several policy and practice implications to our findings. Previous studies have identified an opportunity to modify existing risk communication messages^12,19^ and promote education campaigns.^12,16,18^ Our findings suggest that targeting specific populations and contexts, such as households with children or adjusting messaging for different cultural backgrounds, may be more effective at promoting action, particularly on items that are disaster-specific and unrelated to general consumption and saving patterns. Second, it is important to further study why individuals choose to fulfill some items but not others, such as the role of resource constraints, general awareness, or perceived likelihood of experiencing a disaster. Third, the finding that households with children were more likely to fulfill action-based items suggests that other government programs could integrate preparedness. For example, Medicare and Medicaid have emergency preparedness requirements established for health care practitioners and some specific groups of beneficiaries, such as individuals with disabilities.^32^ There is an opportunity to expand these efforts.

### Limitations

Our study has several limitations. First, conducting a causal or longitudinal analysis was not possible because the data are cross-sectional and the disaster preparedness module changes across the American Household Survey’s data collection cycles. Second, our study sample only includes households that responded to all disaster preparedness questions in the survey. The study sample may be biased toward individuals who are more aware of preparedness actions, thereby overestimating the proportion of US residents who fulfill preparedness items. When we compared the study sample with all survey respondents, respondents who completed all emergency preparedness questions and were thus included in our study were wealthier and more likely to be married and have children. Our population estimates of the proportion of US households that are prepared are likely an overestimate, and it is more appropriate to focus on the adjusted models comparing the household-level predictors of preparedness rather than on absolute levels of preparedness. Additionally, further qualitative research is needed to understand the rationale behind fulfilling each preparedness item or why individuals appear to perceive them differently.

## Conclusions

The findings of this cross-sectional study suggest that the variations in the direction of the associations of household socioeconomic characteristics and resource- and action-based preparedness may indicate that households treat preparedness items differently, such as food and water stockpiles vs having an alternative communication plan. This suggests that households may have different perceptions of which preparedness items are critical or have different capacities to be prepared in different areas depending on the resources and coordination required. As the effects of man-made and natural disasters increase, individuals’ disaster preparedness as a mean of mitigating the effect of emergencies and large-scale disasters are critical to improving the overall emergency response.

## Notes

Supplement. **eTable 1.** Comparison of Full American Household Survey Sample With the Study Population **eTable 2.** Survey Questions for 9 Household Preparedness Items **eTable 3.** Sensitivity Analysis on Overall Preparedness With OLS Specification (Continuous Outcome) and Multinomial Logistic Specification (3-Level Outcome) **eTable 4.** Sensitivity Analysis of Alternative Methods to Specify Income **eTable 5.** Household Characteristics and Fulfillment of Resource- and Action-Based Preparedness Items Using Logistic Regression Weighting for Nonresponse **eTable 6.** Logistic Regression of Adjusted Odds of Overall, Resource-Based, and Action-Based Preparedness Using Logistic Regression Weighting for Nonresponse **eTable 7.** Logistic Regression of Adjusted Odds of Fulfillment of Resource-Based Preparedness Items Using Logistic Regression Weighting for Nonresponse **eTable 8.** Logistic Regression of Adjusted Odds of Fulfillment of Action-Based Preparedness Items Using Logistic Regression Weighting for Nonresponse **eTable 9.** Correlations Among Fulfillment of Specific Preparedness Items
