# Supplementary material for: Association of US Households’ Disaster Preparedness With Socioeconomic Characteristics, Composition, and Region
Source: JAMA Netw Open. 2020 Apr 27;3(4):e206881. doi: 10.1001/jamanetworkopen.2020.6881 (PMC7186859; doi:10.1001/jamanetworkopen.2020.6881)
Supplement: Supplement. — eTable 1. Comparison of Full American Household Survey Sample With the Study Population eTable 2. Survey Questions for 9 Household Preparedness Items eTable 3. Sensitivity Analysis on Overall Preparedness With OLS Specification (Continuous Outcome) and Multinomial Logistic Specification (3-Level Outcome) eTable 4. Sensitivity Analysis of Alternative Methods to Specify Income eTable 5. Household Characteristics and Fulfillment of Resource- and Action-Based Preparedness Items Using Logistic Regression Weighting for Nonresponse eTable 6. Logistic Regression of Adjusted Odds of Overall, Resource-Based, and Action-Based Preparedness Using Logistic Regression Weighting for Nonresponse eTable 7. Logistic Regression of Adjusted Odds of Fulfillment of Resource-Based Preparedness Items Using Logistic Regression Weighting for Nonresponse eTable 8. Logistic Regression of Adjusted Odds of Fulfillment of Action-Based Preparedness Items Using Logistic Regression Weighting for Nonresponse eTable 9. Correlations Among Fulfillment of Specific Preparedness Items [file jamanetwopen-3-e206881-s001.pdf]

Supplementary Online Content

Zamboni LM, Martin EG. Association of US households' disaster preparedness with socioeconomic characteristics, composition, and region. *JAMA Netw Open*. 2020;3(4):e206881. doi:10.1001/jamanetworkopen.2020.6881

eTable 1. Comparison of Full American Household Survey Sample With the Study Population

eTable 2. Survey Questions for 9 Household Preparedness Items

eTable 3. Sensitivity Analysis on Overall Preparedness With OLS Specification (Continuous Outcome) and Multinomial Logistic Specification (3-Level Outcome)

eTable 4. Sensitivity Analysis of Alternative Methods to Specify Income

eTable 5. Household Characteristics and Fulfillment of Resource- and Action-Based Preparedness Items Using Logistic Regression Weighting for Nonresponse

eTable 6. Logistic Regression of Adjusted Odds of Overall, Resource-Based, and Action-Based Preparedness Using Logistic Regression Weighting for Nonresponse

eTable 7. Logistic Regression of Adjusted Odds of Fulfillment of Resource-Based Preparedness Items Using Logistic Regression Weighting for Nonresponse

eTable 8. Logistic Regression of Adjusted Odds of Fulfillment of Action-Based Preparedness Items Using Logistic Regression Weighting for Nonresponse

eTable 9. Correlations Among Fulfillment of Specific Preparedness Items

This supplementary material has been provided by the authors to give readers additional information about their work.

© 2020 Zamboni LM et al. *JAMA Network Open.*

eTable 1. Comparison of Full American Household Survey Sample With the Study Population

**Household Characteristic**	**Study Sample** (N = 16,725)	**Excluded Sample** (N = 16,749)	**American Household Survey** (N = 33,474)
Demographic characteristics of the head of the household			
Female, N (%)	7,622 (45.6)	6,740 (54.6)	14,362 (49.4)
Married, N (%)	11,687 (69.9)	2,373 (19.2)	14,060 (48.4)
Black, N (%)	1,969 (11.8)	2,354 (19.1)	4,323 (14.9)
Hispanic/Latino, N (%)	2,696 (16.1)	1,848 (15.0)	4,544 (15.6)
Age ≥65 years, N (%)	3,579 (21.4)	3,898 (23.3)	7,477 (22.3)
Education of the head of the household			
No high school degree, N (%)	1,772 (10.6)	1,722 (14.0)	3,494 (12.0)
High school degree, N (%)	3,904 (23.3)	2,987 (24.2)	6,891 (23.7)
Some college degree, N (%)	8,497 (50.8)	6,154 (49.9)	14,651 (50.4)
Some graduate degree, N (%)	2,552 (15.3)	1,482 (12.0)	4,034 (13.9)
Additional household characteristics			
Combined household income ($), Median (IQR)	76,500 (76,500)	35,000 (51,320)	55,600 (76,680)
Household has children age <18 years, N (%)	7,163 (42.8)	1,717 (13.9)	8,880 (30.6)
Household has a person with a disability, N (%)	3,533 (21.2)	2,931 (25.4)	6,464 (22.9)
Census region			
South, N (%)	6,469 (38.7)	6,559 (39.2)	13,028 (38.9)
Northeast, N (%)	2,445 (14.6)	2,643 (15.8)	5,088 (15.2)
West, N (%)	4,568 (27.3)	4,538 (27.1)	9,106 (27.2)
Midwest, N (%)	3,243 (19.4)	3,009 (18.0)	6252 (18.7)

Notes: The American Housing Survey included 66,752 housing units, of which 33,474 responded to the topical section on emergency and disaster preparedness. The study sample comprises the housing units that responded to the nine questions on specific preparedness items used for the outcome measures.

© 2020 Zamboni LM et al. *JAMA Network Open.*

eTable 2. Survey Questions for 9 Household Preparedness Items

Preparedness Item	American Housing Survey 2017 Question
Food stockpiles	Does your household have available non-perishable food for 3 days?
Water stockpiles	Does household have available at least 3 gallons or 24 bottles of water per person?
Electric generator	Do you have a generator to provide electricity in case there is a power outage?
Carry-on emergency preparedness kit	Does your household have emergency supplies readily available to take with you if you have to evacuate your home?
Financial resources for evacuation	If you had to evacuate from your town or city to a safe place at least 50 miles away, do you have the financial resources, in terms of savings or available credit card balances, to meet expenses of up to $2,000?
Vehicle available for evacuation	If you had to evacuate from your town or city to a safe place at least 50 miles away do you have enough reliable vehicles to carry all of your household members and a small amount of supplies such as clothes and food?
Alternative communication plan	Do the members of your household have a plan for communicating in the event that cell phone service is disrupted?
Separate evacuation meeting point	If you had to evacuate from your town or city to a safe place at least 50 miles away for at least two weeks, where would you most likely stay during those two weeks?
Plan with financial information	Would you have access to your vital financial information and contact numbers if you had to evacuate your home?

Source: U.S. Department of Housing and Urban Development and U.S. Census Bureau, American Household Survey 2017 codebook, available at https://www.census.gov/data-tools/demo/codebook/ahs/ahsdict.html (accessed March 24, 2020).

© 2020 Zamboni LM et al. *JAMA Network Open.*

eTable 3. Sensitivity Analysis on Overall Preparedness With OLS Specification (Continuous Outcome) and Multinomial Logistic Specification (3-Level Outcome)

**Household Characteristic**	**Logistic Regression** aOR (95% CI)	**OLS Regression** beta (se)	**Multinomial Logit Regression** aOR (95% CI)
Female head of household	0.89 (0.83, 0.96)	-0.13 (0.031)**	0.89 (0.81, 0.98)<br>0.81 (0.73, 0.90)
Married head of household	1.47 (1.36, 1.59)	0.42099 (0.034)**	1.38 (1.25, 1.52)<br>1.86 (1.67, 2.08)
Black head of household	0.99 (0.89, 1.10)	-0.032 (0.047)	0.76 (0.66, 0.87)<br>0.94 (0.80, 1.09)
Hispanic/Latino head of household	0.88 (0.80, 0.97)	-0.16 (0.043)**	0.73 (0.65, 0.83)<br>0.74 (0.65, 0.85)
Head of household aged ≥65 years	1.26 (1.15, 1.39)	0.18 (0.039)**	1.52 (1.33, 1.73)<br>1.53 (1.33, 1.76)
Head of household with high school diploma (reference: no diploma)	1.35 (1.19, 1.52)	0.34 (0.055)**	1.19 (1.02, 1.39)<br>1.45 (1.22, 1.72)
Head of household with some college degree (reference: no diploma)	1.39 (1.23, 1.56)	0.34 (0.052)**	1.36 (1.17, 1.57)<br>1.53 (1.30, 1.81)
Head of household with some graduate degree (reference: no diploma)	1.11 (0.96, 1.28)	0.17 (0.063)*	1.37 (1.13, 1.65)<br>1.25 (1.01, 1.54)
Combined household income ($, logged)	1.13 (1.09, 1.18)	0.14 (0.016)**	1.29 (1.23, 1.35)<br>1.27 (1.21, 1.34)
Household has children <18 years	0.87 (0.81, 0.94)	-0.15 (0.031)**	0.85 (0.77, 0.93)<br>0.78 (0.71, 0.87)
Household has a person with a disability	0.86 (0.79, 0.94)	-0.14 (0.037)**	0.81 (0.73, 0.91)<br>0.77 (0.68, 0.87)
Northeast location (reference: South)	0.77 (0.70, 0.85)	-0.28 (0.044)**	0.92 (0.80, 1.06)<br>0.69 (0.59, 0.80)
West location (reference: South)	0.87 (0.80, 0.95)	-0.11 (0.036)*	0.83 (0.74, 0.94)<br>0.82 (0.72, 0.92)
Midwest location (reference: South)	0.83 (0.76, 0.91)	-0.24 (0.040)**	0.91 (0.81, 1.04)<br>0.70 (0.61, 0.81)

Notes: N=16,527. For the OLS regression, * P<0.01, ** P<0.001. Outcome is overall preparedness, on a 0 to 9 scale. The logistic regression specification, used in the manuscript, represents fulfilling 5 or more (at least half) of items. The ordinary least squares specification uses overall preparedness as a continuous outcome and includes P-values to denote statistical significance. The multinomial logistic regression examines three categories of preparedness (low (0-3 items), medium (4-6 items), and high (7-9 items)), with low as the reference category. For each coefficient, there are two odds ratios corresponding to level 2 versus level 1 (i.e., medium versus low) and level 3 versus level 1 (i.e., high versus low). Abbreviations: adjusted odds ratio (aOR), confidence interval (CI).

© 2020 Zamboni LM et al. *JAMA Network Open.*

eTable 4. Sensitivity Analysis of Alternative Methods to Specify Income

**Household Characteristic**	**Linear**<br>aOR (95% CI)	**Quadratic**<br>aOR (95% CI)	**Quintiles**<br>aOR (95% CI)
Female head of household	0.89 (0.83, 0.96)	0.89 (0.83, 0.95)	0.89 (0.83, 0.95)
Married head of household	1.47 (1.36, 1.59)	1.47 (1.36, 1.59)	1.47 (1.36, 1.58)
Black head of household	0.99 (0.89, 1.10)	0.99 (0.89, 1.10)	0.99 (0.89, 1.10)
Hispanic/Latino head of household	0.88 (0.80, 0.97)	0.88 (0.80, 0.97)	0.88 (0.80, 0.98)
Head of household aged ≥65 years	1.26 (1.15, 1.39)	1.26 (1.15, 1.39)	1.28 (1.16, 1.40)
Head of household with high school diploma (reference: no diploma)	1.35 (1.19, 1.52)	1.35 (1.19, 1.52)	1.34 (1.18, 1.51)
Head of household with some college degree (reference: no diploma)	1.39 (1.23, 1.56)	1.39 (1.23, 1.56)	1.37 (1.22, 1.54)
Head of household with some graduate degree (reference: no diploma)	1.11 (0.96, 1.28)	1.10 (0.95, 1.27)	1.10 (0.95, 1.27)
Combined household income ($, logged)	1.13 (1.09, 1.18)	1.07 (0.81, 1.41)	--
Combined household income, quadratic term ($, logged)	--	1.00 (0.99, 1.02)	--
Combined household income, quintile 2 (reference: quintile 1)	--	--	1.20 (1.08, 1.33)
Combined household income, quintile 3 (reference: quintile 1)	--	--	1.29 (1.16, 1.44)
Combined household income, quintile 4 (reference: quintile 1)	--	--	1.44 (1.28, 1.61)
Combined household income, quintile 5 (reference: quintile 1)	--	--	1.43 (1.27, 1.62)
Household has children <18 years	0.87 (0.81, 0.94)	0.87 (0.81, 0.94)	0.88 (0.81, 0.94)
Household has a person with a disability	0.86 (0.79, 0.94)	0.86 (0.79, 0.94)	0.87 (0.80, 0.94)
Northeast location (reference: South)	0.77 (0.70, 0.85)	0.77 (0.70, 0.85)	0.77 (0.70, 0.85)
West location (reference: South)	0.87 (0.80, 0.95)	0.87 (0.80, 0.95)	0.87 (0.80, 0.95)
Midwest location (reference: South)	0.83 (0.76, 0.91)	0.83 (0.76, 0.91)	0.83 (0.76, 0.91)

Notes: N=16,527. Outcome is fulfilling at least half of the 9 preparedness items (overall preparedness).

© 2020 Zamboni LM et al. *JAMA Network Open.*

eTable 5. Household Characteristics and Fulfillment of Resource- and Action-Based Preparedness Items Using Logistic Regression Weighting for Nonresponse

**Household Characteristic**	**Value**
Demographic characteristics of the head of household	
Female, N (%)	13,447 (49.4)
Married, N (%)	13,773 (50.6)
Black, N (%)	4,158 (15.3)
Hispanic/Latino, N (%)	4,622 (17.0)
Age ≥65 years, N (%)	6,374 (23.4)
No high school degree, N (%)	3,316 (12.2)
High school degree, N (%)	6,535 (24.0)
Some college degree, N (%)	13,536 (49.8)
Some graduate degree, N (%)	3,822 (14.1)
Additional household characteristics	
Combined household income ($), Median (interquartile range)	60,000 (80,640)
Household has children <18 years, N (%)	9,087 (33.4)
Household has a person with a disability, N (%)	5,971 (22.0)
Census region: South, N (%)	10,480 (38.5)
Census region: Northeast, N (%)	4,275 (15.7)
Census region: West, N (%)	7,567 (27.8)
Census region: Midwest, N (%)	4,887 (18.0)
Household availability of resource-based disaster preparedness items	
Vehicle available for evacuation, N (%)	25,712 (94.5)
Food stockpiles, N (%)	22,537 (82.8)
Financial resources for evacuation, N (%)	20,640 (75.7)
Water stockpiles, N (%)	16,246 (59.7)
Carry-on emergency preparedness kit, N (%)	14,646 (53.8)
Electric generator, N (%)	4,602 (16.9)
Household availability of action-based disaster preparedness items	
Plan with financial information, N (%)	21,807 (80.1)
Separate evacuation meeting point, N (%)	9,954 (36.6)
Alternative communication plan, N (%)	7,550 (27.8)
Fulfillment of preparedness criteria	
Overall preparedness, N (%)	17,935 (65.9)
Resource-based preparedness, N (%)	17,054 (62.7)
Action-based preparedness, N (%)	10,718 (39.4)

Notes: N=16,725. For fulfillment of overall preparedness, resource-based items, and action-based items, preparedness is defined as meeting at last half of the items in that category. The study sample is limited to participants who responded to the 9 questions on specific preparedness items. To adjust for possible nonresponse bias, this replicates Table 1 from the manuscript using a logistic regression weighting method.

© 2020 Zamboni LM et al. *JAMA Network Open.*

eTable 6. Logistic Regression of Adjusted Odds of Overall, Resource-Based, and Action-Based Preparedness Using Logistic Regression Weighting for Nonresponse

**Household Characteristic**	**Overall Preparedness** aOR (95% CI)	**Resource-based Preparedness** aOR (95% CI)	**Action-based Preparedness** aOR (95% CI)
Female head of household	0.91 (0.87, 0.96)	0.87 (0.82, 0.91)	0.96 (0.91, 1.01)
Married head of household	1.55 (1.47, 1.64)	1.67 (1.58, 1.77)	1.26 (1.20, 1.33)
Black head of household	0.94 (0.87, 1.01)	0.85 (0.79, 0.92)	1.30 (1.21, 1.40)
Hispanic/Latino head of household	0.83 (0.77, 0.89)	0.92 (0.86, 0.99)	0.92 (0.86, 0.99)
Head of household aged ≥65 years	1.26 (1.18, 1.34)	1.54 (1.44, 1.65)	0.93 (0.87, 0.99)
Head of household with high school diploma (reference: no diploma)	1.18 (1.08, 1.29)	1.28 (1.17, 1.40)	1.04 (0.95, 1.14)
Head of household with some college degree (reference: no diploma)	1.23 (1.13, 1.34)	1.31 (1.21, 1.43)	1.03 (0.95, 1.12)
Head of household with some graduate degree (reference: no diploma)	1.01 (0.91, 1.12)	1.09 (0.98, 1.21)	0.94 (0.85, 1.04)
Combined household income ($, logged)	1.14 (1.12, 1.16)	1.11 (1.09, 1.13)	1.02 (0.99, 1.04)
Household has children <18 years	0.88 (0.83, 0.93)	0.75 (0.71, 0.79)	1.26 (1.19, 1.33)
Household has a person with a disability	0.90 (0.84, 0.96)	0.82 (0.77, 0.87)	1.10 (1.04, 1.17)
Northeast location (reference: South)	0.71 (0.65, 0.76)	0.74 (0.69, 0.80)	0.73 (0.67, 0.78)
West location (reference: South)	0.89 (0.83, 0.95)	0.95 (0.89, 1.02)	0.92 (0.87, 0.98)
Midwest location (reference: South)	0.85 (0.79, 0.91)	0.82 (0.76, 0.88)	0.82 (0.76, 0.88)

Notes: N=16,527. Abbreviations: adjusted odds ratio (aOR), confidence interval (CI). Preparedness is defined by fulfilling at least half of items. The study sample is limited to participants who responded to the 9 questions on specific preparedness items. To adjust for possible nonresponse bias, this replicates Table 2 from the manuscript using a logistic regression weighting method.

© 2020 Zamboni LM et al. *JAMA Network Open.*

eTable 7: Logistic Regression of Adjusted Odds of Fulfillment of Resource-Based Preparedness Items Using Logistic Regression Weighting for Nonresponse

**Household Characteristic**	**Food Stockpile** aOR (95% CI)	**Water Stockpile** aOR (95% CI)	**Electric generator** aOR (95% CI)	**Carry on emergency kit** aOR (95% CI)	**Financial resources for evacuation** aOR (95% CI)	**Evacuation vehicle** aOR (95% CI)
Female head of household	1.07 (1.00, 1.15)	0.95 (0.90, 1.00)	0.82 (0.76, 0.87)	0.90 (0.85, 0.94)	0.73 (0.68, 0.78)	1.06 (0.94, 1.20)
Married head of household	1.27 (1.18, 1.36)	1.34 (1.27, 1.42)	1.83 (1.70, 1.97)	1.22 (1.16, 1.29)	2.03 (1.89, 2.18)	2.53 (2.21, 2.90)
Black head of household	0.71 (0.65, 0.78)	1.15 (1.07, 1.24)	0.48 (0.43, 0.54)	1.30 (1.21, 1.40)	0.44 (0.40, 0.48)	0.34 (0.30, 0.39)
Hispanic/Latino head of household	0.66 (0.61, 0.72)	1.48 (1.38, 1.59)	0.63 (0.57, 0.70)	0.98 (0.92, 1.06)	0.62 (0.57, 0.67)	0.49 (0.43, 0.56)
Head of household aged ≥65 years	1.50 (1.38, 1.64)	1.36 (1.27, 1.45)	1.22 (1.13, 1.32)	1.09 (1.02, 1.16)	2.10 (1.92, 2.30)	1.07 (0.94, 1.23)
Head of household with high school diploma (reference: no diploma)	1.18 (1.06, 1.32)	0.89 (0.81, 0.97)	1.20 (1.06, 1.35)	1.06 (0.97, 1.16)	1.69 (1.53, 1.86)	1.07 (0.94, 1.23)
Head of household with some college degree (reference: no diploma)	1.11 (1.01, 1.24)	0.79 (0.73, 0.86)	1.08 (0.96, 1.22)	0.99 (0.92, 1.08)	2.73 (2.48, 2.99)	2.01 (1.73, 2.33)
Head of household with some graduate degree (reference: no diploma)	0.97 (0.85, 1.10)	0.63 (0.57, 0.70)	0.72 (0.62, 0.83)	0.86 (0.77, 0.95)	10.76 (8.98, 12.88)	2.32 (1.83, 2.93)
Combined household income ($, logged)	1.06 (1.04, 1.09)	0.97 (0.95, 0.99)	1.10 (1.07, 1.13)	1.05 (1.03, 1.07)	1.46 (1.43, 1.50)	1.20 (1.16, 1.24)
Household has children <18 years	0.98 (0.92, 1.06)	0.75 (0.71, 0.79)	0.76 (0.71, 0.83)	0.90 (0.85, 0.95)	0.68 (0.63, 0.73)	1.08 (0.95, 1.23)
Household has a person with a disability	1.05 (0.97, 1.14)	0.89 (0.83, 0.94)	1.19 (1.10, 1.29)	0.96 (0.90, 1.02)	0.54 (0.50, 0.58)	0.57 (0.50, 0.64)

© 2020 Zamboni LM et al. *JAMA Network Open.*

**Household Characteristic**	**Food Stockpile** aOR (95% CI)	**Water Stockpile** aOR (95% CI)	**Electric generator** aOR (95% CI)	**Carry on emergency kit** aOR (95% CI)	**Financial resources for evacuation** aOR (95% CI)	**Evacuation vehicle** aOR (95% CI)
Northeast location (reference: South)	0.86 (0.78, 0.95)	0.85 (0.79, 0.91)	1.01 (0.91, 1.11)	0.64 (0.60, 0.69)	1.05 (0.95, 1.15)	0.43 (0.38, 0.50)
West location (reference: South)	0.83 (0.77, 0.90)	1.14 (1.07, 1.21)	0.67 (0.62, 0.73)	0.90 (0.85, 0.96)	1.15 (1.06, 1.25)	0.87 (0.75, 1.01)
Midwest location (reference: South)	0.99 (0.90, 1.09)	0.81 (0.76, 0.87)	1.05 (0.96, 1.15)	0.66 (0.62, 0.71)	1.07 (0.97, 1.17)	1.10 (0.93, 1.32)

Notes: N=16,527. Abbreviations: adjusted odds ratio (aOR), confidence interval (CI). The study sample is limited to participants who responded to the 9 questions on specific preparedness items. To adjust for possible nonresponse bias, this replicates Table 3 from the manuscript using a logistic regression weighting method.

© 2020 Zamboni LM et al. *JAMA Network Open.*

eTable 8. Logistic Regression of Adjusted Odds of Fulfillment of Action-Based Preparedness Items Using Logistic Regression Weighting for Nonresponse

**Household Characteristic**	**Alternative communication plan** aOR (95% CI)	**Alternative meeting location** aOR (95% CI)	**Plan with financial information** aOR (95% CI)
Female head of household	0.96 (0.90, 1.01)	0.97 (0.92, 1.02)	0.99 (0.92, 1.05)
Married head of household	1.18 (1.11, 1.25)	1.23 (1.16, 1.30)	1.25 (1.17, 1.34)
Black head of household	1.57 (1.46, 1.70)	1.21 (1.13, 1.31)	0.91 (0.83, 0.99)
Hispanic/Latino head of household	1.07 (0.99, 1.16)	0.90 (0.84, 0.97)	0.68 (0.62, 0.73)
Head of household aged ≥65 years	0.90 (0.84, 0.97)	0.84 (0.79, 0.90)	1.17 (1.08, 1.27)
Head of household with high school diploma (reference: no diploma)	1.05 (0.96, 1.15	1.04 (0.95, 1.14)	1.22 (1.11, 1.35)
Head of household with some college degree (reference: no diploma)	0.96 (0.88, 1.05)	1.04 (0.96, 1.14)	1.49 (1.35, 1.63)
Head of household with some graduate degree (reference: no diploma)	0.90 (0.80, 1.00)	0.96 (0.86, 1.07)	1.55 (1.37, 1.76)
Combined household income ($, logged)	0.95 (0.93, 0.97)	1.01 (0.99, 1.03)	1.26 (1.23, 1.29)
Household has children <18 years	1.11 (1.04, 1.18)	1.27 (1.2, 1.34)	0.95 (0.88, 1.02)
Household has a person with a disability	1.07 (1.00, 1.15)	1.13 (1.07, 1.21)	0.83 (0.77, 0.90)
Northeast location (reference: South)	0.76 (0.70, 0.82)	0.73 (0.67, 0.79)	0.77 (0.70, 0.84)
West location (reference: South)	1.06 (0.99, 1.14)	0.98 (0.92, 1.04)	0.74 (0.69, 0.81)
Midwest location (reference: South)	0.77 (0.71, 0.84)	0.94 (0.88, 1.01)	0.82 (0.75, 0.90)

Notes: N=16,527. Abbreviations: adjusted odds ratio (aOR), confidence interval (CI). The study sample is limited to participants who responded to the 9 questions on specific preparedness items. To adjust for possible nonresponse bias, this replicates Table 4 from the manuscript using a logistic regression weighting method.

© 2020 Zamboni LM et al. *JAMA Network Open.*

eTable 9. Correlations Among Fulfillment of Specific Preparedness Items

Item	Water stockpile	Electric generator	Carry on emergency kit	Financial resources for evacuation	Evacuation vehicle	Alternative communication plan	Alternative meeting location	Plan with financial information
Food stockpile	0.273	0.108	0.253	0.144	0.088	0.098	0.118	0.180
Water stockpile		0.100	0.347	0.088	0.042	0.225	0.184	0.119
Electric generator			0.117	0.119	0.053	0.044	0.061	0.077
Carry on emergency kit				0.111	0.059	0.331	0.305	0.246
Financial resources for evacuation					0.209	0.032	0.052	0.227
Evacuation vehicle						0.020	0.048	0.106
Alternative communication plan							0.522	0.143
Alternative meeting location								0.160

Notes: Pearson correlation coefficients. P-values are not provided because with such a large sample, many associations are statistically significant although not meaningful qualitatively.

© 2020 Zamboni LM et al. *JAMA Network Open.*
